# A comparative study of the disease burden attributable to asbestos in Brazil, China, Kazakhstan, and Russia between 1990 and 2019

**DOI:** 10.1186/s12889-022-14437-6

**Published:** 2022-11-03

**Authors:** Jieyuan Chen, Chunfei Wang, Jinyu Zhang, Ting Zhang, Hongsen Liang, Songsong Mao, Haifeng Li, Zhaojun Wang

**Affiliations:** 1 Department of Anesthesiology, Guangdong Provincial People’s Hospital, Guangdong Academy of Medical Sciences, Guangzhou, China; 2 Endoscopy Center, The Seventh Affiliated Hospital, Sun Yat-Sen University, Shenzhen, China; 3 Department of Thoracic Surgery, The Seventh Affiliated Hospital, Sun Yat-Sen University, Shenzhen, China

**Keywords:** Asbestos, DALYs, Disease burden, Global Burden of Disease, Estimated annual percentage change

## Abstract

**Background:**

Brazil, China, Kazakhstan, and Russia are the main asbestos-producing countries, and all forms of asbestos are carcinogenic to humans. The objective of this study was to estimate the disease burden attributable to asbestos between 1990 and 2019 in major producing countries, including Brazil, China, Kazakhstan, and Russia.

**Methods:**

Age-standardized mortality rates (ASMR) and age-standardized disability-adjusted life year (DALY) rates (ASDR) of disease burden attributable to asbestos by country, age, and sex were extracted from the Global Burden of Disease 2019. Percentage change and estimated annual percentage change (EAPC) were used to assess the trends of ASDR and ASMR of disease burden attributable to asbestos between 1990 and 2019.

**Results:**

Asbestos-related diseases were highly heterogeneous across Global, Brazil, China, Kazakhstan, and Russia. There was a downward trend in ASMR and ASDR of diseases burden related to asbestos globally. The age-specific mortality rate of disease attributable to asbestos increased in men and women, although it decreased in women aged 85–89, the highest age-specific mortality rate were observed in age 95 + group in men [162.14 (95% UI: 103.76–215.45)] and women [30.58 (95% UI: 14.83–44.33)] per 100 000 population, respectively. Tracheal, bronchus, and lung (TBL) cancer was the leading cause of death and DALYS attributable to asbestos between 1990 and 2019 globally and in Brazil, China, Kazakhstan, and Russia. China had the highest percentage change (73.31%) and EAPC [3.41 (95% CI: 2.75–4.08)] in ASMR related to exposure to asbestos in men, with the highest percentage change (73.31%) and EAPC [3.41 (95% CI: 2.75–4.08)] in ASDR in men.

**Conclusions:**

The ASMR and ASDR of disease burden attributable to asbestos decreased between 1990 and 2019 globally. TBL cancer was the leading cause of death and DALYs attributable to asbestos between 1990 and 2019. There has been an increasing trend in mortality and DALYs globally, especially in older men. The burden of disease attributable to asbestos is increasing in China, especially in men.

**Supplementary Information:**

The online version contains supplementary material available at 10.1186/s12889-022-14437-6.

## Background

Occupational carcinogens cause a considerable disease burden globally and at the national level [1, 2]. In 2016, 349,000 deaths and 7.2 million disability-adjusted life years (DALYs) were attributed to occupational carcinogens. Asbestos is responsible for the greatest number of occupational cancer deaths [3]. Although the health risks of asbestos were recognized early, the ban was implemented gradually and late [4]. In 2007, the World Health Assembly Resolution 60.26 called for a global campaign to eliminate asbestos-related diseases [5]. To date, 67 of 195 countries around the world have banned asbestos [6]. Asbestos is still being produced and exported in major producing countries such as Brazil, China, Kazakhstan, and Russia [7].

Asbestos refers to six naturally occurring fibrous minerals: amosite, actinolite, anthophyllite, chrysotile, crocidolite, and tremolite [8], and it is used for insulation in buildings and as an ingredient in a number of products. Exposure to asbestos causes cancer of the lung, larynx, ovaries, and mesothelioma, as well as asbestosis. According to the World Health Organization (WHO), 125 million people worldwide are exposed to asbestos at work. At least 90,000 people die each year from asbestos-related lung cancer, mesothelioma, and asbestosis, according to global estimates [5].

Brazil, China, Kazakhstan, and Russia account for more than 90% of the world's total asbestos production [9]. In this study, we present results from the Global Burden of Disease (GBD) 2019 and provide an assessment of current trends of disease burden attributable to asbestos in Brazil, China, Kazakhstan, and Russia between 1990 and 2019. These countries need to be made aware of the burden of asbestos to implement a ban as soon as possible.

## Methods

### Data sources

The data used in this study were obtained from the GBD 2019 globally and in countries between 1990 and 2019 (http://ghdx.healthdata.org/gbd-results-tool). Relevant data were extracted to analyze the status of disease burden attributable to asbestos globally and in Brazil, China, Kazakhstan, and Russia. There is a hierarchy of risk factors in the GBD 2019, Risk factors in Level 1 are behavioural, environmental, occupational, and metabolic; risk factors in Level 2 include 20 risks; and risk factors in Level 3 include 52 risks [10], when selecting occupational exposure to asbestos, there are five diseases in level 3 [tracheal, bronchus, and lung (TBL) cancer, pneumoconiosis, ovarian cancer, mesothelioma, and larynx cancer].

### Descriptive study

Age-standardized rates of mortality and disability-adjusted life years (DALYs) of all causes attributable to asbestos between 1990 and 2019 were collected for Global, Brazil, China, Kazakhstan, and Russia. Number of deaths, age-specific DALYs, and mortality rates attributable to asbestos were extracted by age and sex in 2019 for Global. To investigate the differences in disease burden related to exposure to asbestos, age-standardized DALY rate (ASDR) and age-standardized mortality rate (ASMR) were analyzed for five different diseases related to exposure to asbestos between 1990 and 2019 globally and in Brazil, China, Kazakhstan, and Russia. Prism software (GraphPad Prism 8, USA) was used for data presentation. Data were summarized using R software (version 4.1.2).

### Statistical analysis

Percentage change and estimated annual percentage change (EAPC) were used to assess the trends of ASDR and ASMR attributable to asbestos between 1990 and 2019. EAPC based on the age standardized rates was used to reflect the temporal trends of ASMR and ASDR [11]. EAPC was calculated as EAPC = 100 × (exp(β)—1), where β is the regression coefficient of the linear model [12]. Calculation of EAPC included the 95% confidence interval (95% CI). R software was used to calculate the percentage change and EAPC.

## Results

### Disease burden attributable to asbestos by country, sex, and age

**Fig. 1:**
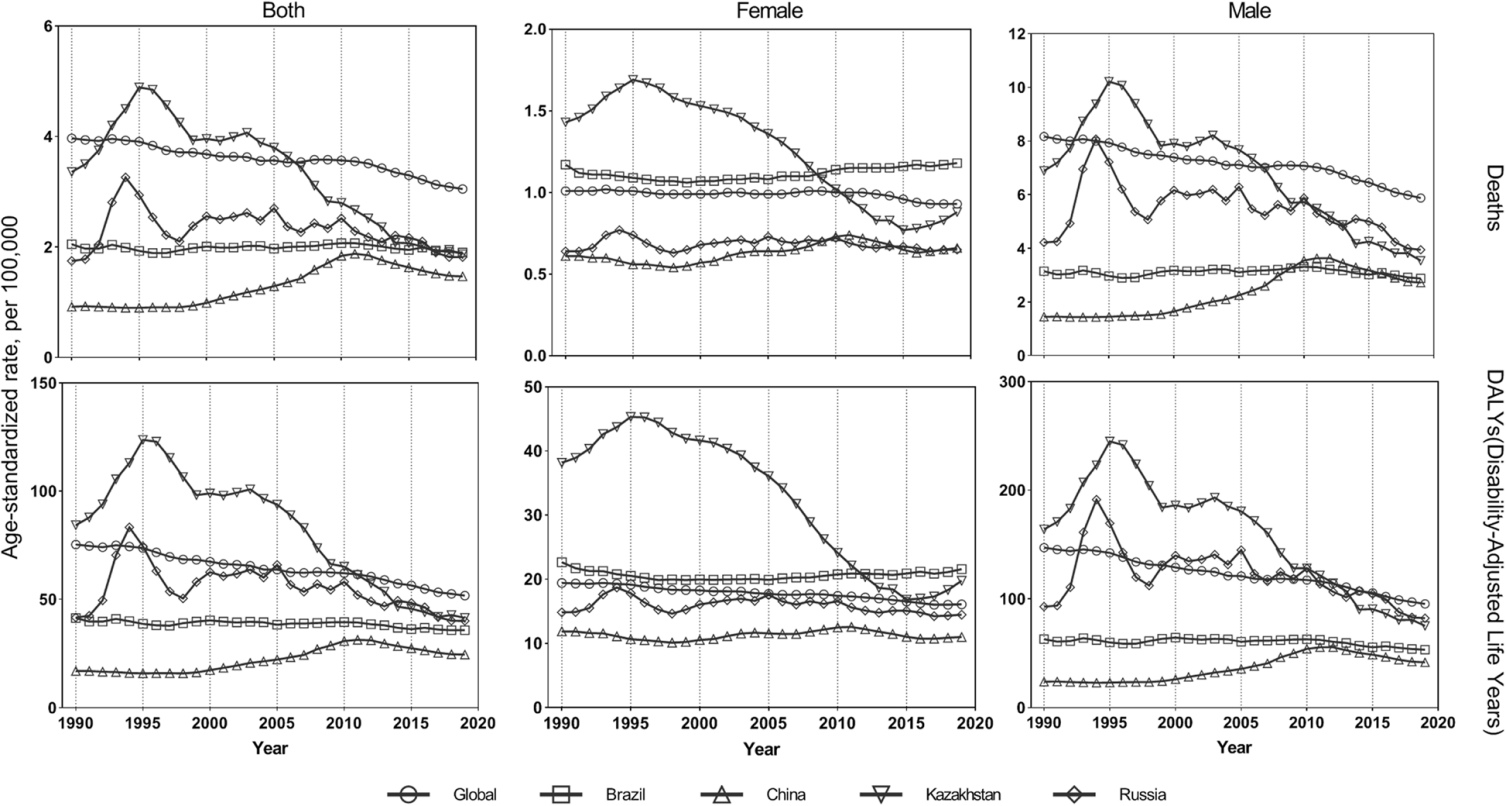
The age-standardized rates of mortality and DALYs of all causes attributable to asbestos from 1990 to 2019 for global, Brazil, China, Kazakhstan, and Russia. DALYs, disability-adjusted life years

The disease burden attributable to asbestos was highly heterogeneous across Global, Brazil, China, Kazakhstan, and Russia. The ASMR and ASDR of disease attributable to asbestos showed a downward trend globally. Kazakhstan had the highest ASMR and ASDR of disease attributable to asbestos in both men and women. The ASMR and ASDR of disease attributable to asbestos increased between 1990 and 1995 in Kazakhstan in both sexes and in men, followed by a decrease; the trend in women was slightly different with an increase in 2015. In Kazakhstan, the highest ASMR and ASDR of disease attributable to asbestos in both sexes were 4.89 (95% UI: 3.01–7.28) per 100 000 population and 123.75 (95% UI: 75.7–192.09) per 100 000 population, respectively, in 2015 (Table S1). The ASMR and ASDR of disease attributable to asbestos increased between 1990 and 1994 in Russia, followed by a decreasing trend, although the decrease in 2019 was not significant compared with 1990. In Russia, the highest ASMR [3.26 (95% UI: 2.2–4.54)] and ASDR [83.25 (95% UI: 55.62–116.47)] of disease attributable to asbestos were observed in both sexes in 1994 per 100 000 population (Table S2). Brazil showed a relatively stable trend between 1990 and 2019 in both men and women; however, the ASMR and ASDR of disease attributable to asbestos was higher in Brazil than in China, Kazakhstan, and Russia in women. A comparison of global, Brazil, Kazakhstan, and Russia trends showed that the ASMR and ASDR of disease attributable to asbestos was lowest in China. Similar trends were observed in men and in both sexes (Fig. 1).

**Fig. 2:**
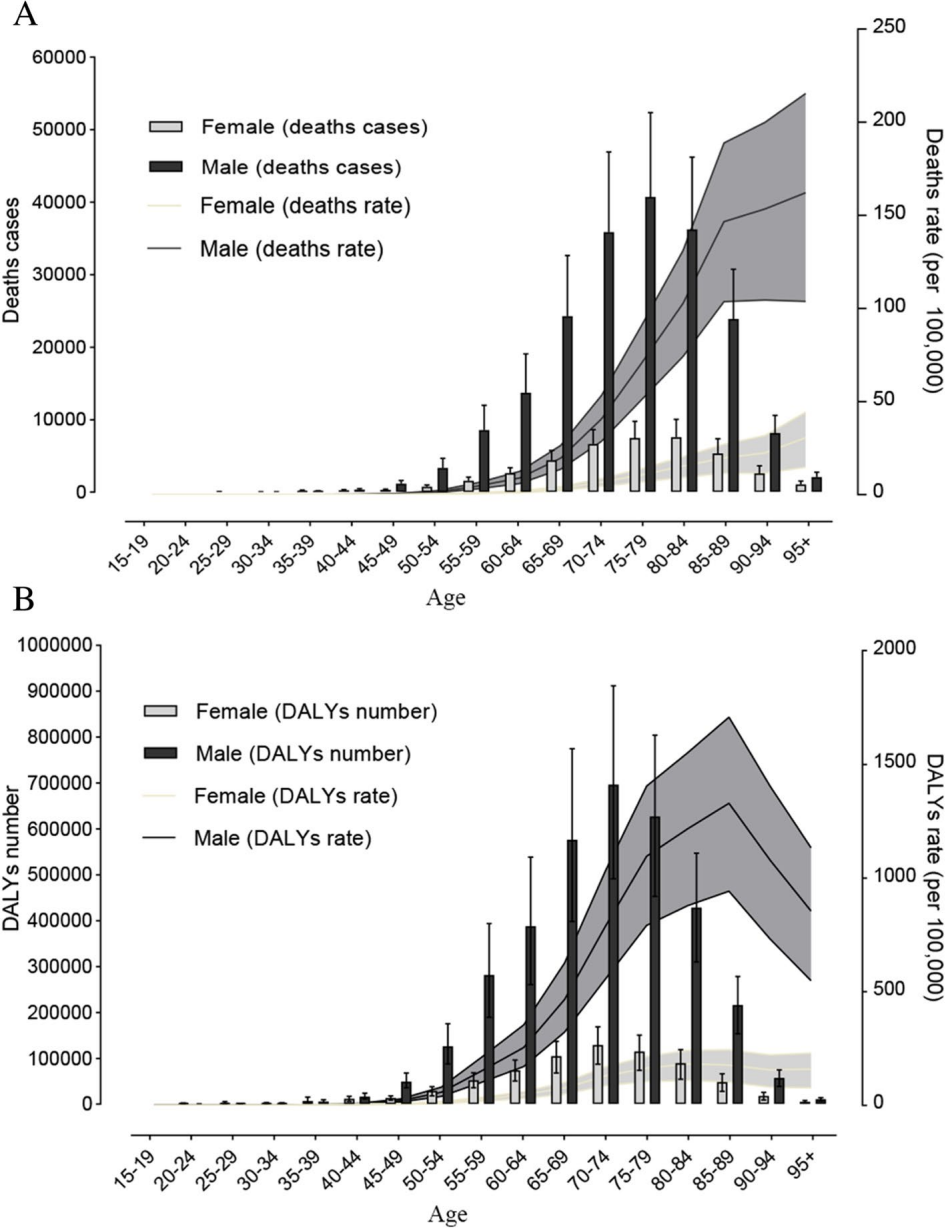
Age-specific counts and rates of deaths (**A**), and DALYs (**B**) of all causes exposure to asbestos by sex in 2019 for Global. DALYs, disability-adjusted life-years

There was one peak in the number of age-specific deaths in women and men (age 75–79 years in men and 80–84 years in women), the number of age 75–79 years in men and age 80–84 years in women were 40,734.55 (95% UI: 29,416.25–52,360.55) and 7572.94 (95% UI: 4578.47–10,047.57), respectively (Table S3); the number of deaths was higher in men than in women (Fig. 2A). The age-specific mortality rate was higher in men than in women. The age-specific mortality rate increased exponentially before age 85–89 years in men, and then increased linearly with a slow increase in men, the highest age-specific mortality rate of disease attributable to asbestos were observed in age 95 + group in men [162.14 (95% UI: 103.76–215.45)] and women [30.58 (95% UI: 14.83–44.33)] per 100 000 population, respectively (Table S3). There has been a lower increase in age-specific mortality rate in women (Fig. 2A). There was one peak in the number of age-specific DALYs in women and in men (age 70–74 years in men and women), the number of age 70–74 years in men and women were 696,841.12 (95% UI: 491,470.36–911,945.71) and 128,978.36 (95% UI: 87,076.39–168,457.49), respectively (Table S4); the number of DALYs was higher in men than in women. The age-specific DALY rate increased exponentially before age 75–79 years in men, then increased linearly, peaking at age 85–89 [1329.08 (95% UI: 942.22–1708.09)] followed by a decreasing trend (Table S4). The DALY rate was lower in women, showing a slow rise before age 80–84 years, then becoming stable (Fig. 2B).

### Disease burden attributable to asbestos by cause

**Fig. 3:**
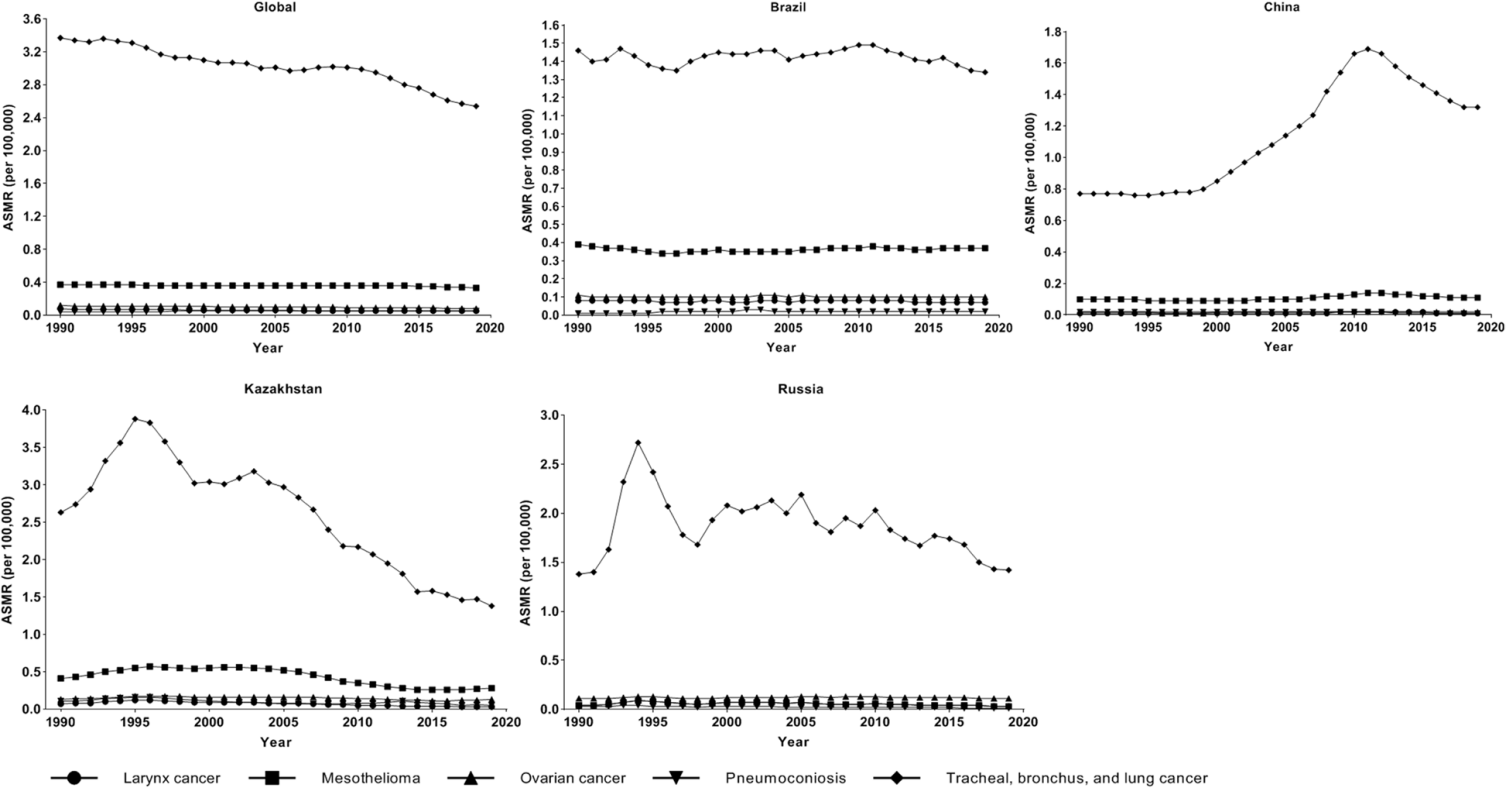
The age-standardized mortality rates of causes exposure to asbestos in Global, Brazil, China, Kazakhstan, and Russia from 1990 to 2019

**Fig. 4:**
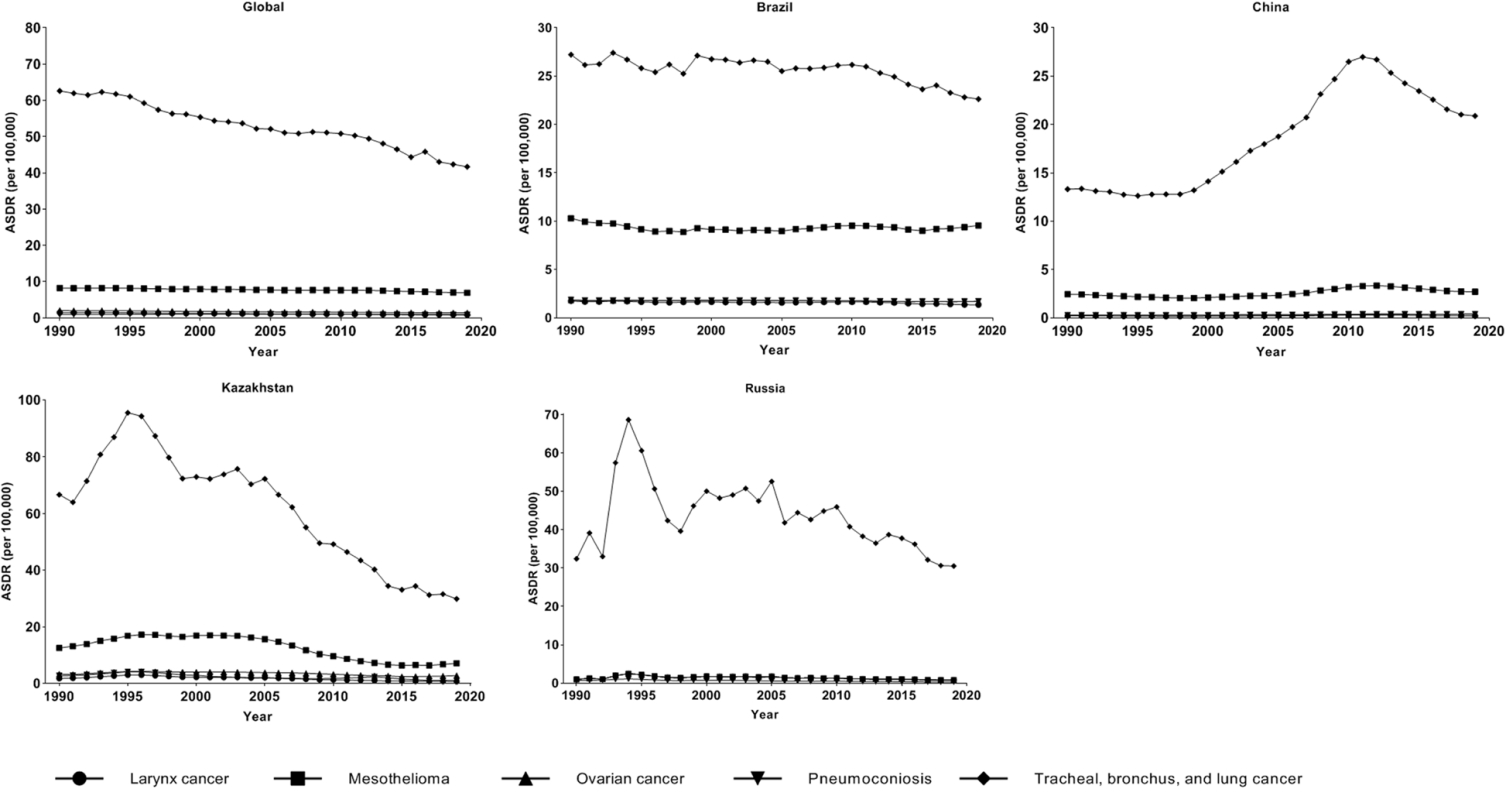
The age-standardized DALYs rates of causes exposure to asbestos in Global, Brazil, China, Kazakhstan, and Russia from 1990 to 2019. DALYs, disability-adjusted life years

In GBD 2019, five diseases were attributable to asbestos in level 3 causes, including TBL cancer, pneumoconiosis, ovarian cancer, mesothelioma, and larynx cancer. TBL cancer was the leading cause of death and DALYs attributable to asbestos between 1990 and 2019 globally and in Brazil, China, Kazakhstan, and Russia, followed by mesothelioma; the remaining three diseases posed a relatively small burden (Figs. 3 and 4). The ASMR and ASDR of TBL cancer attributable to asbestos showed a downward trend from 1990 to 2019 globally; a stable trend in the ASMR and ASDR of TBL cancer attributable to asbestos was observed between 1990 and 2019 in Brazil. The ASMR and ASDR of TBL cancer attributable to asbestos were stable before 1999, then increased gradually and peaked in 2011, followed by a decrease in China (Figs. 3 and 4), the ASMR and ASDR of TBL cancer attributable to asbestos were 1.69 (95% UI: 1.1–2.36) and 26.99 (95% UI: 17.35–38.24) in China in 2011 (Table S5 and S6). The ASMR and ASDR of TBL cancer attributable to asbestos increased rapidly before 1994, then showed a downward trend in Kazakhstan and Russia (Figs. 3 and 4), the ASMR and ASDR of TBL cancer attributable to asbestos were 2.72 (95% UI: 1.67–3.99) and 68.64 (95% UI: 41.71–101.69) in Russia in 1994 (Table S5 and S6). After 1998, the ASMR and ASDR of TBL cancer attributable to asbestos began to fluctuate in Russia, and the decrease was not significant between 1998 and 2019. The ASMR and ASDR of TBL cancer attributable to asbestos were higher in Kazakhstan than in Brazil, China, and Russia. The ASMR and ASDR of mesothelioma attributable to asbestos were higher in Brazil and Kazakhstan than in China and Russia (Figs. 3 and 4).

### Disease burden attributable to asbestos

In 2019, the ASMR and ASDR of disease attributable to asbestos were 3.05 [95% uncertainty interval (UI): 2.29–3.82] per 100,000 population and 51.77 (95% UI: 38.71–65.65) per 100,000 population, respectively, decreasing by 23.17% and 31.27%, respectively, compared with 1990 in global. EAPC was -0.79 (95% CI: -0.89 – -0.69) for death and -1.23 (95% CI: -1.32 – -1.14) for DALYs between 1990 and 2019 (Tables 1 and 2). Russia, and Kazakhstan were ranked the top two in the ASMR and ASDR of disease attributable to asbestos in 2019 in men. Among women, Brazil ranked first in the ASMR and ASDR of disease attributable to asbestos in 2019. The ASMR of disease attributable to asbestos was 0.92 (95% UI: 0.62–1.38) per 100 000 population in 1990 and 1.47 (95% UI: 0.98–2.13) per 100 000 population in 2019 in China, with percentage change being 59.78%. The ASDR of disease attributable to asbestos was 16.99 (95% UI: 11.32–25.4) per 100 000 population in 1990 and 24.55 (95% UI: 16.26–35.87) per 100 000 population in 2019 in China, with percentage change being 44.50%. China had the highest percentage change (87.59%) and EAPC [3.69 (95% CI: 3.01–4.37)] in ASMR related to exposure to asbestos in men, whereas Kazakhstan had the highest decrease in percentage change (-48.62%) and EAPC [-3.09 (95% CI: -3.74 – -2.44)] in ASMR related to exposure to asbestos in men. China had the highest percentage change (73.31%) and EAPC [3.41 (95% CI: 2.75–4.08)] in ASDR related to exposure to asbestos in men, whereas Kazakhstan had the highest decrease in percentage change (-54.06%) in men and EAPC [-3.69 (95% CI: -4.32 – -3.04)] in women in ASDR related to exposure to asbestos.

**Table 1:** The age-standardized mortality rate of disease attributable to asbestos in 1990 and 2019, and its percentage change and estimated annual percentage change by sex for Global, Brazil, China, Kazakhstan, and Russia, from 1990 to 2019

location	Gender	ASMR_1990 (95% UI)	ASMR_2019 (95% UI)	Percentage change	EAPC (95% CI)
Global	Both	3.97 (2.97–4.98)	3.05 (2.29–3.82)	-23.17%	-0.79 (-0.89 to -0.69)
	Female	1.01 (0.72–1.34)	0.93 (0.6–1.21)	-7.92%	-0.22 (-0.30 to -0.14)
	Male	8.17 (5.87–10.49)	5.88 (4.18–7.59)	-28.03%	-0.99 (-1.09 to -0.88)
Brazil	Both	2.05 (1.56–2.6)	1.9 (1.44–2.39)	-7.32%	-0.01 (-0.12 to 0.10)
	Female	1.17 (0.76–1.57)	1.18 (0.78–1.56)	0.85%	0.22 (0.11 to 0.34)
	Male	3.15 (2.13–4.27)	2.88 (1.98–3.92)	-8.57%	0.02 (-0.15 to 0.19)
China	Both	0.92 (0.62–1.38)	1.47 (0.98–2.13)	59.78%	2.80 (2.24 to 3.36)
	Female	0.61 (0.37–1.03)	0.66 (0.36–0.96)	8.20%	0.69 (0.41 to 0.97)
	Male	1.45 (0.82–2.46)	2.72 (1.59–4.3)	87.59%	3.69 (3.01 to 4.37)
Kazakhstan	Both	3.36 (2.03–5.47)	1.87 (1.15–2.87)	-44.35%	-2.97 (-3.61 to -2.32)
	Female	1.43 (0.38–4.31)	0.88 (0.31–1.94)	-38.46%	-2.84 (-3.32 to -2.36)
	Male	6.89 (4.23–10.27)	3.54 (2.12–5.24)	-48.62%	-3.09 (-3.74 to -2.44)
Russia	Both	1.75 (1.22–2.37)	1.82 (1.19–2.55)	4%	-0.54 (-1.17 to 0.09)
	Female	0.64 (0.44–0.94)	0.65 (0.43–0.94)	1.56%	-0.10 (-0.32 to 0.12)
	Male	4.22 (2.63–6.15)	3.95 (2.28–5.89)	-6.40%	-0.88 (-1.54 to -0.21)

*UI* Uncertainty interval, *CI* Confidence interval, *EAPC* Estimated annual percentage change, *ASMR* Age standardized mortality rates

**Table 2:** The age-standardized DALYs rate of disease attributable to asbestos in 1990 and 2019, and its percentage change and estimated annual percentage change by sex for Global, Brazil, China, Kazakhstan, and Russia, from 1990 to 2019

location	Gender	ASDR_1990 (95% UI)	ASDR_2019 (95% UI)	Percentage change	EAPC (95% CI)
Global	Both	75.32 (55.94–95.25)	51.77 (38.71–65.65)	-31.27%	-1.23 (-1.32 to -1.14)
	Female	19.41 (13.48–25.78)	16.08 (10.67–20.68)	-17.16%	-0.66 (-0.71 to -0.62)
	Male	147.2 (105.28–190.56)	95.45 (67.75–124.58)	-35.16%	-1.40 (-1.50 to -1.31)
Brazil	Both	41.39 (31.66–52.46)	35.75 (27.96–44.63)	-13.63%	-0.34 (-0.44 to -0.23)
	Female	22.64 (14.03–31.62)	21.56 (13.63–28.71)	-4.77%	0.00 (-0.14 to 0.14)
	Male	63.14 (43.55–84.9)	53.48 (38.04–71.22)	-15.30%	-0.37 (-0.54 to -0.19)
China	Both	16.99 (11.32–25.4)	24.55 (16.26–35.87)	44.50%	2.46 (1.92 to 3.01)
	Female	11.88 (7.04–20.76)	11.01 (6.01–16.3)	-7.32%	0.11 (-0.15 to 0.36)
	Male	24.13 (13.75–41.4)	41.82 (24.5–66.62)	73.31%	3.41 (2.75 to 4.08)
Kazakhstan	Both	84.22 (50.11–143.01)	41.53 (25.84–63.95)	-50.69%	-3.52 (-4.23 to -2.80)
	Female	38.17 (8.79–124.6)	19.74 (7.08–46.89)	-48.28%	-3.69 (-4.32 to -3.04)
	Male	163.9 (102.02–243.12)	75.3 (43.87–112.68)	-54.06%	-3.54 (-4.25 to -2.83)
Russia	Both	41.68 (29.01–56.95)	40.09 (25.87–56.54)	-3.81%	-0.98 (-1.69 to -0.27)
	Female	14.85 (10.09–22.34)	14.46 (9.74–21.93)	-2.63%	-0.35 (-0.63 to -0.07)
	Male	92.94 (57.96–134.95)	82.29 (47.35–122.73)	-11.46%	-1.23 (-1.98 to -0.48)

*UI* Uncertainty interval, *CI* Confidence interval, *ASDR* Age standardized DALYs rates, *EAPC* Estimated annual percentage change, *DALYs* Disability-adjusted life years

## Discussion

The International Commission on Occupational Health calls for a global ban on the mining, sale, and use of all forms of asbestos to eliminate asbestos-related diseases [13]. The production and use of asbestos has been reduced or banned in many countries, although some countries continue to produce or consume the material. The estimated global production of asbestos in 2020 was 1.2 million tons; the top four producing countries are Russia (790,000 tons), Kazakhstan (210,000 tons), China (100,000 tons), and Brazil (60,000 tons). In recent years, worldwide consumption of asbestos fiber is estimated to have decreased from roughly two million tons in 2010 to about one million tons per year [7]. China and Russia are the biggest users. Most of the deaths in high-income areas are caused by carcinogens, and asbestos-related cancers account for approximately 80% of all cancer deaths in these areas [14]. In low and middle income countries, exposure to asbestos still occurs, which may have a devastating effect if current and future exposure is not controlled.

Asbestos is used as insulation in buildings and in a variety of consumer products, such as water supply lines, roofing shingles, gaskets, and clutches and brake linings [5]. It is expected that asbestos-cement products, such as pipes, corrugated roofing tiles, and wall panels, will continue to be the world's dominant asbestos market. Even after demolitions, renovations, or destruction of asbestos-containing materials, asbestos can still be found in buildings and in the environment. This legacy asbestos may also be a risk to the general population [4, 15]. There are thousands of deaths linked to asbestos exposure in homes.

The present findings indicate that the burden of disease attributable to asbestos is on a downward trend globally. The number of deaths are higher in men than in women, and the age-specific mortality rate is higher in men than in women. This may be related to a male predominance among workers. The mortality rates are high in the elderly, which is consistent with previous results. Because asbestos-related diseases have long latency periods, the number of asbestos-related deaths will not decrease immediately after the use of asbestos is discontinued [5]. Even in countries that banned asbestos in the early 1990s, asbestos-related diseases continue to increase.

In this study, we showed that TBL cancer was the leading cause of death and DALYs attributable to asbestos between 1990 and 2019 globally and in Brazil, China, Kazakhstan, and Russia, followed by mesothelioma. Although the ASMR and ASDR of TBL cancer attributable to asbestos decreased in recent years, the decrease is not obvious in China and Russia. China and Russia should pay special attention to screening for TBL cancer.

Although China’s disease burden attributable to asbestos is relatively low compared with that in Kazakhstan and Russia, China showed the highest percentage change and EAPC in ASMR and ASDR related to exposure to asbestos between 1990 and 2019, especially in men. According to China’s Ministry of Industry and Information Technology, the mining and processing of chrysotile requires workers to wear protective clothing since 2014 [16]; however, chrysotile is not completely banned. Asbestos should also be restricted and eventually banned by the Chinese government. The ASMR and ASDR of disease attributable to asbestos is stable in Brazil. Although asbestos was banned nationwide in Brazil in November 2017, the government of the state of Goias passed a law that permitted asbestos mining in the state for export in July 2019, and asbestos is still produced in Brazil. It is predicted that China and Brazil will face a public health crisis on an unprecedented scale caused by the use of asbestos [17]. Kazakhstan was the fourth-largest producer of asbestos, although the ASMR and ASDR of disease attributable to asbestos have been decreasing in Kazakhstan. The WHO started to support Kazakhstan's efforts to eliminate asbestos-related diseases in 2011. However, the government of Kazakhstan has never banned asbestos, and diseases are estimated to be under-reported by a large margin. The global economy still encourages asbestos production and use in Russia [18]. Uralasbest runs the world’s largest operating chrysotile asbestos mine in Russian Federation [19]. Although Russia does not have the highest disease burden of asbestos in 2019, these are particularly troubling result. In fact, according to the WHO, mining and exploitation of minerals is the most effective method to eliminate diseases attributable to asbestos. It is time to ban asbestos completely around the world.

The present study had several limitations. First, the risk cannot be quantified because epidemiological studies lack accurate exposure information. Second, the GBD results are based on the estimates obtained by combining the system dynamics model and the statistical model, which does not constitute real observation data, and the estimated results could be inaccurate. Furthermore, the prevalence of asbestos may be underestimated in low income countries with poor health supervision systems.

## Conclusion

This study assessed the disease burden attributable to asbestos in Brazil, China, Kazakhstan, and Russia. Although the burden of disease attributable to asbestos declined globally, it remains highly heterogeneous in Brazil, China, Kazakhstan, and Russia. TBL cancer is the leading cause of death and DALYS attributable to asbestos. There has been an increasing trend in mortality and DALYs in China, especially in older men. We call for a global ban on asbestos, especially in Brazil, China, Kazakhstan, and Russia.

## Supplementary Information

**Additional file 1:** **Table S1.** The ASMR and ASDR of disease attributable to asbestos in Kazakhstan.

**Additional file 2:** **Table S2.** The ASMR and ASDR of disease attributable to asbestos in Russia.

**Additional file 3:** **Table S3.** Age-specific counts of deaths of all causes exposure to asbestos in 2019 for Global.

**Additional file 4:** **Table S4.** Age-specific counts of DALYs of all causes exposure to asbestos in 2019 for Global.

**Additional file 5: Table S5.** The ASMR of causes exposure to asbestos in Global, Brazil, China, Kazakhstan, and Russia.

**Additional file 6:** **Table S6.** The ASDR of causes exposure to asbestos in Global, Brazil, China, Kazakhstan, and Russia.

## Data Availability

The datasets generated and/or analysed during the current study are available in the Global Health Data Exchange (GHDx) repository, [http:// ghdx. healt hdata. org/ gbd- resul ts- tool].
